# Treatment of Carbuncle

**Published:** 1877-05-20

**Authors:** J. H. Dibrell

**Affiliations:** Little Rock, Arkansas


### TREATMENT OF CARBUNCLE.

BY J. H. DIBRELL, JR., M. D., of Little Rock, Arkansas.

The use of collodion, in conjunction with carbolic acid, has yielded, in my practice, and in that of several medical friends whom I have requested to try it, such satisfactory, and it is believed unusual, results, as to induce the belief in its superiority over other modes of treatment.

Carbolic acid, in this affection, has been recommended by several writers, with much favor, one of whom claims to have aborted the disease by the early hypodermic use of the acid, in a case that promised to assume formidable proportions. My plan is as follows:—When the carbuncle is seen early, to puncture it, and with a camels hair pencil, or small pointed stick, introduce into the opening thus made the pure and undiluted acid. If the disease has made greater progress, and one or more small acne-like pustules have made their appearance on the tumor, these are carefully opened, which can be done without causing pain, and the acid introduced at each opening, as before indicated. The effect of the acid when first applied, especially if it touch a denuded surface, is to produce a sharp, stinging pain, which is, however, of but momentary duration. The next effect is local anæsthesia, and the patient is, for a time, perhaps hours, free from pain.

Carbolic acid possessing in a notable degree anæsthetic, antiseptic and caustic properties, would seem to be peculiarly adapted to the treatment of the disease under consideration, which is usually attended with great pain, sloughing, and an intolerable odor. Its use in my hands has certainly seemed to diminish the pain, correct the odor and to arrest the sloughing process with much promptitude.

After the acid has been applied collodion should be several times painted over the carbuncle, and beyond it, a few lines, on the uninflamed skin. *All the openings are to be left free,* in order to give egress to discharges. Each layer or film of the collodion should be allowed to dry before another is put on. This dressing may be renewed once daily, and the collodion previously applied, if partially detached, should be peeled off before a new application is made. If the part on which the carbuncle makes its appearance be covered with hair, this should be cleanly shaved off, otherwise the collodion will be difficult to remove and at the same time cause considerable pain.

It is interesting to watch the collodion as it contracts upon the diseased tissues. The skin, previously red and swollen, will in a few minutes be seen, through the transparent gun cotton, to have become pale and depressed, as the pressure gradually empties the engorged capillaries. If the disease is advanced, and sloughs have become partly separated, they are not unfrequently forced out or brought so near the openings as to be readily detached with scissors. This pressure does not give rise to pain, but on the contrary, generally affords much relief to the suffering patient. The application of collodion in this disease has other advantages. It limits the extent of the disease in decreasing the vascularity of the part, and in this way lessens the inflammatory action going on, and probably also prevents the absorption of pus. It also protects the surrounding skin from contact with the discharges which, as is well known, is capable of producing, if not an extension of the disease, numerous small boils, which are of themselves an exceedingly annoying complication. Should however any such pustules or boils be formed in the course of the disease, they can be cut short by touching them with carbolic acid. After the carbuncle has been treated with the acid and collodion, it should be protected from contact with the clothing, by covering it over with a piece of old linen or cotton cloth, saturated with sweet oil, or spread with carbolic acid cerate.—*Medical and Surgical Reporter.*

